# Microbiota variations in *Culex nigripalpus* disease vector mosquito of West Nile virus and Saint Louis Encephalitis from different geographic origins

**DOI:** 10.7717/peerj.6168

**Published:** 2019-01-09

**Authors:** Dagne Duguma, Michael W. Hall, Chelsea T. Smartt, Mustapha Debboun, Josh D. Neufeld

**Affiliations:** 1 Florida Medical Entomology Laboratory, Institute of Food and Agricultural Sciences, University of Florida, Vero Beach, FL, USA; 2 Mosquito and Vector Control Division, Harris County Public Health, Houston, TX, USA; 3 Faculty of Graduate Studies, Dalhousie University, Halifax, NS, Canada; 4 Department of Biology, University of Waterloo, Waterloo, ON, Canada

**Keywords:** Mosquitoes, Insect vectors, Biogeography, Viral disease, Microbiome, Culex

## Abstract

Although mosquito microbiota are known to influence reproduction, nutrition, disease transmission, and pesticide resistance, the relationship between host-associated microbial community composition and geographical location is poorly understood. To begin addressing this knowledge gap, we characterized microbiota associated with adult females of *Culex nigripalpus* mosquito vectors of Saint Louis Encephalitis and West Nile viruses sampled from three locations in Florida (Vero Beach, Palmetto Inland, and Palmetto Coast). High-throughput sequencing of PCR-amplified 16S rRNA genes demonstrated significant differences among microbial communities of mosquitoes sampled from the three locations. Mosquitoes from Vero Beach (east coast Florida) were dominated by uncultivated *Asaia* sp. (*Alphaproteobacteria*), whereas microbiota associated with mosquitoes collected from two mosquito populations at Palmetto (west coast Florida) sites were dominated by uncultured *Spironema culicis* (*Spirochaetes*), *Salinisphaera hydrothermalis* (*Gammaproteobacteria*), *Spiroplasma* (*Mollicutes*), uncultured *Enterobacteriaceae*, Candidatus Megaira (*Alphaproteobacteria*; *Rickettsiae*), and *Zymobacter* (*Gammaproteobacteria*). The variation in taxonomic profiles of *Cx. nigripalpus* gut microbial communities, especially with respect to dominating taxa, is a potentially critical factor in understanding disease transmission and mosquito susceptibility to insecticides among different mosquito populations.

## Background

*Culex nigripalpus* Theobald is a primary vector of Saint Louis Encephalitis (SLE) virus in North America and also an important vector for other arboviruses, such as West Nile virus (WNV) in southeastern United States (Kopp et al., 2013; Day, Tabachnick & Smartt, 2015). It occurs widely in various habitats, including the transitional zone between salt and freshwater coastal habitats, irrigated agricultural sites, and engineered treatment wetlands (O’Meara & Evans, 1983; Darsie & Ward, 2005). This species typically represents the dominant *Culex* colonizer of newly formed aquatic habitats (Duguma et al., 2017a). Genetic variations within populations of *Cx. nigripalpus* vary in time and space (Nayar, Knight & Munstermann, 2002), including tolerance to organophosphate-based adulticides that occurs in some populations (Boike et al., 1989; Shin & Smartt, 2016). Although variations of genetic factors may largely be responsible for variations in susceptibility to pathogens and pesticide tolerances, the role of non-genetic factors, such as microbial symbionts found in different *Cx. nigripalpus* populations, is not well understood.

The microbiota of mosquitoes can help provide nutrition for successful development (Mitraka et al., 2013; Coon et al., 2014), influence pathogen transmission (Glaser & Meola, 2010; Finney, Kamhawi & Wasmuth, 2015; Zink et al., 2015; Shaw et al., 2016; Novakova et al., 2017), and impact susceptibility to pesticides (Patil et al., 2013; De Almeida et al., 2017; Dada et al., 2018). Some mosquito-associated microorganisms have also been implicated in degrading insecticides, such as organophosphates, and may enhance mosquito tolerance to pesticides (De Almeida et al., 2017; Soltani et al., 2017; Dada et al., 2018). Dada et al. (2018) reported a dominance of bacteria associated with organophosphate-degrading enzymes in organophosphate resistant malaria vector mosquito, *Anopheles albimanus* Wiedemann suggesting a link between insecticide resistance and microbiota composition. Mosquitoes devoid of microbiota (using antibiotics) were more susceptible to *Bacillus thuringiensis* compared to mosquitoes that contain natural microflora (Patil et al., 2013). The success of a *Wolbachia*-based biocontrol program, which is dependent on the success of colonization of *Wolbachia* strains into mosquito populations that lack those strains, has also been reported to be potentially limited by larval environmental factors (Hancock et al., 2016). Possible effects of native microbial communities present in various larval habitats, and ingested by larval mosquitoes, naturally occurring in different mosquito populations may influence the success of this type of control program. *Wolbachia* and other microbiota in mosquitoes varied across seasons in WNV mosquito vectors and *Wolbachia* infections were found negatively correlated with WNV in these mosquitoes (Novakova et al., 2017). Microbiota in naturally occurring mosquitoes are known to differ from laboratory-reared mosquitoes (Duguma et al., 2015), and are likely to influence mosquito vector competence both seasonally and locally (Novakova et al., 2017). Spatial and temporal environmental variations influence the microbiota of insects, including fruit flies and mosquitoes (Corby-Harris et al., 2007), and can alter susceptibility to pathogen infections (Akorli et al., 2016; Novakova et al., 2017).

To better investigate the roles of microbiota in *Cx. nigripalpus*, first it is important to understand biogeographical patterns for microbial communities within unique *Cx. nigripalpus* field populations. By sampling adult female mosquitoes from three different geographical locations in Florida, the goal of this research was to identify core microbial taxa, despite geographical distance, in addition to microorganisms that are uniquely distributed spatially. In a previous study, mosquito microbiota composition in general did not vary despite differences in environmental variables such as the amount of nutrients available in the developmental habitat (Duguma et al., 2017a). Microbiota from mosquitoes originating from mesocosms placed in the same geographic location with high and low nutrient concentration in water column did not differ significantly (Duguma et al., 2015, 2017a). As a result, here, we hypothesized that adult *Cx. nigripalpus* mosquito populations sampled from different geographic locations would not differ in their gut microbiota. Characterizing the microbiota associated with *Cx. nigripalpus* will help identify potential symbionts and known pathogens that can be used to develop novel approaches for mosquito control, predict variations in susceptibility to different pesticides, and monitor pathogen infections among different populations.

## Materials and Methods

### Sampling

**Figure 1:**
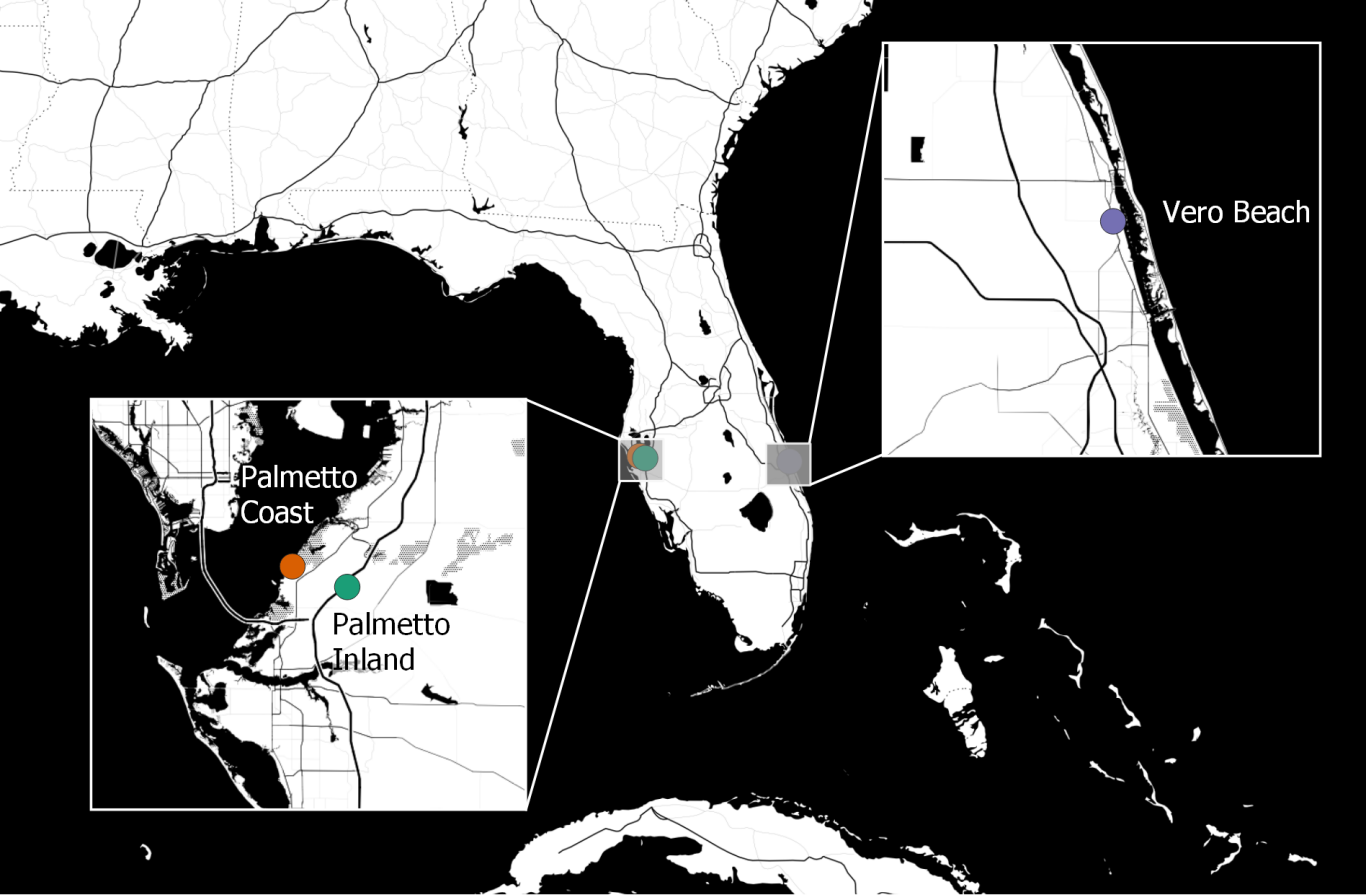
Geographic locations of *Cx. nigripalpus* mosquito sampling sites for microbiome study. Geographic locations of three mosquito sampling sites (two sampling sites from Palmetto sites (Palmetto Coast and Palmetto Inland), and one sampling site from Vero Beach) in Florida, USA. Image source: Map tiles by Stamen Design, under CC BY 3.0 license. Data by OpenStreetMap, under ODbL license (http://maps.stamen.com). Map generated with QGIS v3.2.3-Bonn (QGIS Development Team, 2018).

Adult females of *Cx. nigripalpus* were sampled from three coastal habitats of Florida in autumn 2015, including the Institute of Food and Agricultural Sciences Florida Medical Entomology Laboratory (FMEL; University of Florida, Gainesville, FL, USA) outdoor aquatic mosquito experiment station in Vero Beach (east coast Florida; 27.5876 N, −80.3735 W) and two sites from Palmetto located on the west coast of Florida (Fig. 1). The two Palmetto sites are referred to as “Palmetto Coast” (27.6405 N, −82.5527 W) and “Palmetto Inland” (27.6279 N, −82.4968 W). Although both Palmetto locations are over 200 km west of the Vero Beach site, Palmetto Coast is located about one km from coastal habitats of the Gulf of Mexico and Palmetto Inland is located about six km southwest of the Palmetto Coast site and is closer to residential areas.

Samples were collected in October 2015 and December 2015 from Vero Beach and Palmetto sites, respectively. Adult mosquitoes at the Vero Beach site were sampled following a previously described procedure (Duguma et al., 2017a). Late instar larvae were taken from four outdoor aquatic mesocosms located at the FMEL campus and placed into four modified BioQuip mosquito-rearing jars (BioQuip, Inc., Rancho Dominguez, CA, USA) for adult emergence where adults were collected as soon as they emerged. The mesocosms were constructed using cattle tanks (volume = 378 L) and placed in the field environment surrounded by oak trees to simulate natural breeding habitats for *Culex* species including *Cx. nigripalpus*. The trees surrounding mesocosms provided continuous supply of organic substrates, source microbes, and mosquitoes. The modified BioQuip mosquito-rearing jars float in the mesocosms with the larvae placed in the mesh screen in the water column allowing mosquito larvae access to microbial food sources (Duguma et al., 2017a). *Culex nigripalpus* larvae were identified using the keys before placed in BioQuip mosquito-rearing jars (Cutwa & O’Meara, 2006). Upon adult emergence, female adults were euthanized and placed in freezer until DNA extraction. Together, four samples (one sample per mesocosm) were collected from Vero Beach site. Vero Beach mosquitoes did not feed on blood or sugar post emergence. The mosquitoes from Palmetto sites were collected using CDC Miniature light traps (John W. Hock Company, Gainesville, FL, USA) with dry ice as bait. These mosquitoes were collected from Manatee County Mosquito Control District operational sites, ensuring exposure of at least parents of the analyzed mosquitoes to insecticides. However, no attempt was made to detect presence of pesticide resistance in mosquitoes. A total of four trap samples, two from each of Palmetto Inland and Palmetto Coast were collected. Mosquitoes were identified to species and sorted by their sexes microscopically, kept in 95% ethanol, and stored in a freezer until DNA extraction and further processing.

Together, 16 mosquito samples, including four from the Vero Beach site and 12 from the two Palmetto sites, representing east and west coast mosquito populations were analyzed for their microbial community profiles. A sample consisted of a pool of three intact individual female adult mosquitoes used for DNA extraction. The mosquitoes from Vero Beach were captured and euthanized following eclosion. However, we have no information on the age of mosquitoes from Palmetto sites because they were captured in CDC light traps. Although mosquitoes collected and used from Palmetto sites were not blood-fed ones, we have not excluded the possibility that they may have fed on vertebrates in their life time.

### DNA extraction, PCR, and sequencing

Surface sterilization by 95% ethanol followed by triple rinsing using molecular biology grade UltraPure water (Quality Biological, Inc., Gaithersburg, MD, USA), followed by air drying was performed before DNA extraction. The DNA extraction, PCR, and sequencing procedures were described in previous studies (Bartram et al., 2011; Duguma et al., 2015, 2017a). The DNA from the whole body of mosquitoes was extracted using DNeasy Blood and Tissue Kit following the manufacturer’s protocol (Qiagen, Inc., Valencia, CA, USA) under a laminar flow hood. We only extracted DNA from adult females to focus on them and identify bacterial symbionts associated with females and likely to be maternally inherited. Amplicons of approximately 460 base pairs were generated by PCR of V3 and V4 regions of 16S rRNA genes using Pro341F (5′-CCTACGGGNBGCASCAG-3′) and Pro805R (5′-GACTACNVGGGTATCTAATCC-3′), targeting both bacteria and archaea (Takahashi et al., 2014). Amplicons from each of the samples were tagged with six-base barcodes, amplified using Illumina-specific primers, pooled, and subjected to 250-base paired-end sequencing (Reagent Kit V2, 500) on a MiSeq (Illumina Inc., San Diego, CA, USA).

### Data analyses

Sequence analysis was performed using QIIME2 (2018.6 release) with additional analyses performed using R in Jupyter notebooks. Divisive amplicon denoising algorithm 2 (version 1.6.0) was used to generate the amplicon sequence variants (ASVs) from the raw sequences and detect and remove chimeric sequences (Callahan et al., 2016). The consensus sequences for the ASVs were classified with a scikit-learn Naïve Bayes classifier trained against the most recent SILVA 16S rRNA gene reference (release 132) database (Quast et al., 2012). Sequences were rarefied to the lowest number of sequences per sample (31,183) for alpha diversity analyses. Non-metric multidimensional scaling based on the weighted and unweighted (i.e., binary and non-binary) Bray–Curtis dissimilarities was performed using R package “vegan” version 2.5-2 (Oksanen et al., 2007). From the same package, the multi-response permutation procedure (MRPP) was used to test differences among sample groups. Faith’s phylogenetic diversity (Faith, 1992) was computed with QIIME2 by using the generated phylogeny as input (Katoh & Standley, 2013) and phylogenies were created by FastTree version 2.1.10 (Price, Dehal & Arkin, 2010). Faith’s phylogenetic diversity (Faith, 1992) alpha-diversity measure was computed with QIIME2, using the generated phylogeny as input. Differences in alpha diversity measured by phylogenetic diversity were determined using Kruskal–Wallis followed by Dunn’s post hoc test with Benjamini–Hochberg multiple hypothesis test correction. Upset plots were used to determine overlaps of ASVs and sequences from mosquitoes sampled from the three locations (Conway, Lex & Gehlenborg, 2017). The relative abundance plot was generated using the full data set, whereas the ordinations plots were based on rarefied data.

Samples from this study were sequenced along with samples analyzed by previous publications (Duguma et al., 2017a, 2017b), and 24 non-template negative controls were used in the same sequence run. Downstream analysis of the sequences revealed that our biological field samples were distinct from the very few microbial taxa associated with negative controls, suggesting that the mosquito sample data we analyzed were not affected by laboratory or reagent contamination. In addition, the non-template samples had fewer sequences (maximum of 16,000 sequences in negative control samples, versus a minimum of 34,286 sequences in field samples). The negative controls were dominated by *Facklamia*, *Staphylococcus epidermidis*, *Vibrio metschnikovii*, and *Actinomycetales*, and were removed from further analysis and interpretation.

The sequence dataset generated for this study was submitted to the European Bioinformatics Institute under project accession number PRJEB24029. Data and notebooks are available on GitHub (https://github.com/mwhall/PeerJ_Culex_nigripalpus). Scripts for this publication have been archived in release 1.0 (DOI 10.5281/zenodo.1484552).

## Results

To explore the microbiota of wild adult female *Cx. nigripalpus* mosquitoes sampled from three field locations in Florida (Fig. 1), we generated a total sequence dataset of 1,583,222 16S rRNA gene sequences that clustered into 1,482 ASVs, including 1,198 bacteria, 257 unclassified ASVs, 25 Eukaryotes, and two archaea. Together, the bacterial ASVs were affiliated with 22 unique phyla. *Proteobacteria* was the dominant phylum associated with adult female *Cx. nigripalpus*, with site-specific average relative abundances ranging between 35.3% and 99.7% among samples from the three locations (Table 1). In samples that contained lower relative proportions of *Proteobacteria*, ASVs associated with *Spirochaetes* and *Tenericutes* dominated *Cx. nigripalpus* microbiota at >10% relative abundance.

**Table 1:** Bacterial phyla associated with adult female *Cx. nigripalpus*.

Phylum	Palmetto Inland	Palmetto Coast	Vero Beach
*Proteobacteria*	65.32	35.27	99.72
*Spirochaetes*	15.88	30.60	0.00
*Tenericutes*	10.39	13.41	0.00
*Actinobacteria*	2.92	5.66	0.01
*Bacteroidetes*	2.73	3.55	0.00
*Firmicutes*	0.66	4.42	0.04
*Cyanobacteria*	0.06	0.67	0.17
*Acidobacteria*	0.19	0.63	0.00
*Epsilonbacteraeota*	0.51	0.02	0.03
*Patescibacteria*	0.12	0.30	0.00
*Gemmatimonadetes*	0.01	0.26	0.00
Unclassified *Bacteria*	0.14	0.75	0.00
Unassigned	0.70	3.91	0.01
Others	0.37	0.81	0.01

**Note:**

Bacterial phyla associated with adult female *Cx. nigripalpus*. Average taxonomic proportions (%) for microbiota identified in sampled adult female *Cx. nigripalpus* from three sites in Florida. Only bacterial phyla representing an average of ≥0.1% from at least one location are shown. “Unclassified” refers to proportion of sequences that could not be classified to bacterial phyla whereas “Unassigned” category refers to sequences that could not be classified to *Bacteria* domain. “Others” include summed proportions of all other ASVs in 14 bacterial phyla, Eukarya and Archaea.

**Figure 2:**
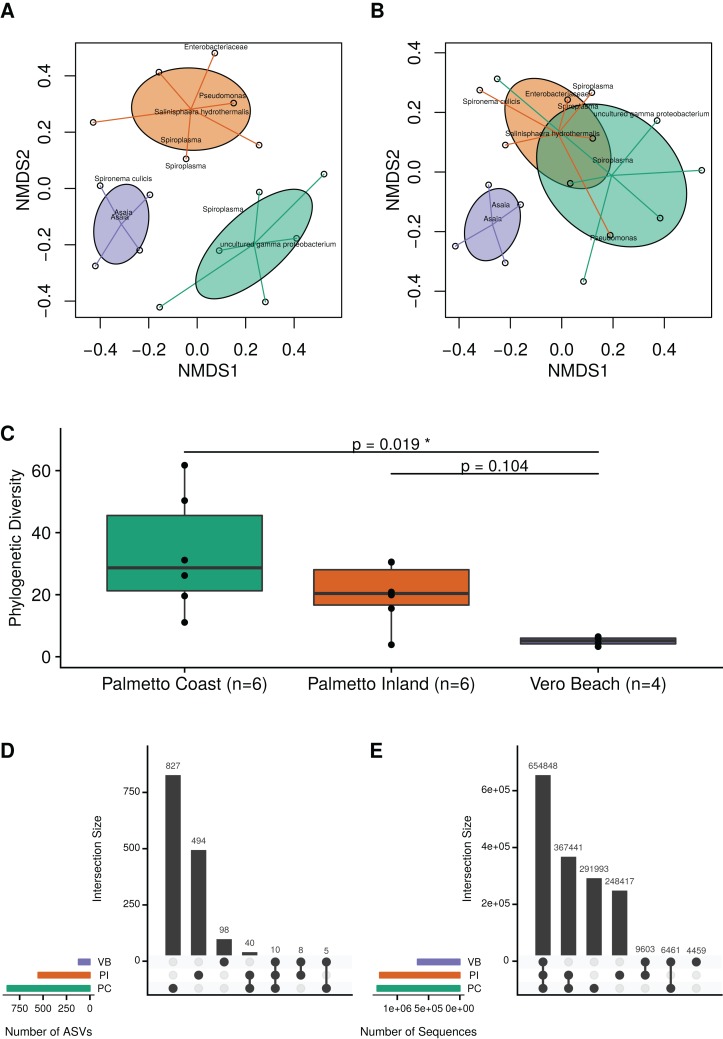
Microbiota diversity associated with female adult *Cx. nigripalpus* mosquitoes. Microbiota diversity associated with female adult *Cx. nigripalpus* mosquitoes collected from three Florida locations. Non-metric multidimensional scaling (NMDS) of microbiota from wild female *Cx. nigripalpus* mosquitoes collected from Vero Beach and two Palmetto sites (Palmetto Coast and Palmetto Inland) of Florida using the weighted Bray–Curtis (A) and non-weighted Bray–Curtis (B) dissimilarity measures. These ordinations revealed microbial communities in mosquitoes differed by sites (MRPP test; *A* = 0.19; *p* = 0.001 for weighted; *A* = 0.06; *p* = 0.001 for unweighted). (C) Phylogenetic diversity plots of microbiota from wild *Cx. nigripalpus* mosquitoes collected from three Florida locations: Vero Beach, Palmetto Inland, and Palmetto Coast. Dunn’s test *p*-values comparing the PD medians between sites. Only Palmetto Coast and Vero Beach were significantly different (*p* = 0.02). Upset plots showing the number of ASVs shared between sites (D) and the intersection of ASVs by site, weighted by their sequence count (E).

Ordination analysis using a weighted Bray–Curtis distance measure revealed consistent differences in microbial communities among mosquitoes collected from the three sample site locations (Fig. 2A; MRPP test; *A* = 0.19; *p* = 0.001). Similarly, an ordination based on unweighted Bray–Curtis (i.e., Sørensen–Dice) distances revealed that microbiota from wild *Cx. nigripalpus* mosquitoes collected from Vero Beach and two Palmetto sites (Palmetto Inland and Palmetto Coast) differed significantly, but with a smaller effect size than the abundance-weighted measure (Fig. 2B; MRPP test; *A* = 0.06; *p* = 0.001). The mosquitoes from the Palmetto Coast site contained significantly higher microbial diversity than the Vero Beach site (Fig. 2C; Dunn’s test; *p* = 0.02). The Palmetto Inland mosquitoes had a higher median phylogenetic diversity than the Vero Beach mosquitoes, but this difference was not statistically significant (Fig. 2C; Dunn’s test; *p* = 0.104).

At the ASV level of resolution, there were no ASVs found in all samples. The majority of ASVs were observed only in one location (Fig. 2D). However, there were 10 ASVs observed in all three sites (Fig. 2D) which accounted for 654,848 sequences and represented 41.4% of all sequences (Fig. 2E). This included an abundant ASV of *Spironema culicis*, two abundant ASVs of *Asaia*, and seven relatively low abundance (<0.1%) but nonetheless frequently present ASVs, including *Thorsellia, Arcobacter, Rubritepida*, and uncultured *Erysipelotrichaceae*.

**Figure 3:**
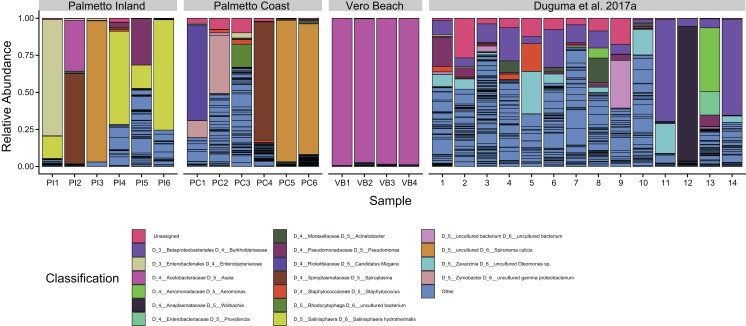
Dominant bacterial amplicon sequence variants (ASVs) associated with adult *Cx. nigirpalpus* mosquitoes. Dominant (found in >10% relative abundance in at least one sample) taxa found in female *Cx. nigripalpus* adult mosquitoes collected from Vero Beach and two Palmetto sites (Palmetto Inland and Palmetto Coast) of Florida, as well as those collected from adult *Cx. nigripalpus* mosquitoes at Vero Beach reported in Duguma et al. (2017a). Only adult data from Duguma et al. (2017a) was included for comparison of taxa with this study. The “Others” category include summed proportions of all other ASVs, including unclassified bacteria. Black lines delineate ASVs.

One ASV classified to *Asaia* sp. of the *Acetobacteraceae* was the dominant taxon from the Vero Beach site, accounting for 56.1–81.0% of all sequences (Fig. 3). This ASV, while dominating the Vero Beach mosquitoes, was detected in very low abundance in one sample from each of the Palmetto Coast and Palmetto Inland sites (<1%). Overall, *Asaia* ASVs accounted for between 96.8% and 99.3% of all Vero Beach sequences, mostly due to a second large *Asaia* ASV. The microbiota in mosquitoes collected from the Palmetto sites were dominated by *Spironema culicis*, *Salinisphaera hydrothermalis*, *Spiroplasma*, unidentified ASVs of *Enterobacteriaceae*, Candidatus Megaira (*Rickettsiaceae*)*, Zymobacter*, *Pseudomonas*, and several unidentified *Rhizobiaceae* ASVs (Fig. 3).

## Discussion

Many factors including sex, age, size, geographic location, and other physiological status of mosquitoes have been shown to influence mosquito-associated microbiota (Osei-Poku et al., 2012; Zouache et al., 2010; Buck et al., 2016; Minard et al., 2018). Our study corroborated these previous findings and indicated that factors associated with different geographic locations might have impacted microbiota composition variations observed in adult female *Cx. nigripalpus* collected from different geographic locations. It is possible that the larval environment of the adult mosquitoes collected from the different sites and the physiological statuses of the mosquitoes including age and parity may have contributed to this variation (Akorli et al., 2016). The mosquitoes from Vero Beach were collected from a relatively similar larval site; however, we are uncertain of the specific larval habitats of samples collected from the two Palmetto sites. The samples from Vero Beach were collected from mesocosms located in one location. In contrast, because mosquitoes from Palmetto sites were trapped as adults in CDC light traps, we could not exactly describe their larval developmental origin. While our study has not accounted for the possible effect of age, size, and other physiological statuses, distinct microbiota in the mosquitoes might reflect differences in environmental factors associated with larval developmental sites or geographic locations. Using baseline data from this study, future research will help disentangle whether variations in microbiota among samples observed in this study may be due to factors such as local microclimatic differences in mosquito development or physiological status.

*Proteobacteria* dominated microbiota in *Cx. nigripalpus* mosquitoes (this study; Duguma et al., 2017a) similar to studies on different mosquito species (Duguma et al., 2013; Minard, Mavingui & Moro, 2013; Dada et al., 2014). It is currently unknown whether the dominance of *Proteobacteria* members may have some inhibitory effects whether through competition inside the host to other bacterial taxa, but our studies show that *Cx. nigripalpus* samples that contained higher proportion of *Proteobacteria* contained lower proportion of *Spirochaetes*, *Tenericutes*, and *Firmicutes*.

The ASVs classifying to the alphaproteobacterial genus *Asaia* dominated all of the Vero Beach samples (Fig. 3). The sequences identified to *Asaia* in this study were the same sequences identified to *Swaminathania* in previous studies (Duguma et al., 2017a, 2017b), but were not observed in such high relative abundances in the previous studies. *Asaia* spp. symbionts have previously been linked to several medically important mosquitoes, including *Anopheles* malaria vectors, with some suggesting a critical role for the *Asaia* bacteria in the complete development of *Anopheles* mosquito species (Chouaia et al., 2012; Mitraka et al., 2013) and *Aedes* mosquitoes (Favia et al., 2007; Damiani et al., 2010). These bacteria were also detected in four species of the *Cx. pipiens* Linaeus complex, including *Cx. quinquefasciatus* Say (De Freece et al., 2014). *Asaia* species have been reported to outcompete and impede the proliferation of *Wolbachia* in both laboratory-reared and field-collected mosquitoes (Hughes et al., 2014; Rossi et al., 2015; Novakova et al., 2017). Because of the *Asaia* association with both laboratory and field-collected mosquitoes, and reported anti-*Plasmodium* properties, this bacterial species has been considered for paratransgenic control of mosquitoes (Wilke & Marrelli, 2015). Our finding of variations of *Asaia* occurrence in *Cx. nigripalpus* vector populations warrants further investigation, including sequencing the genome of this bacterial strain. Further investigation is warranted to determine the proliferation of *Asaia* taxa in some mosquito samples and whether its dominance is correlated with the scarcity of *Wolbachia* in *Cx. nigripalpus* (Duguma et al., 2017a, 2017b), as has been suggested for *Anopheles* mosquitoes (Rossi et al., 2015).

A gammaproteobacterial genus *Salinisphaera* was detected at >10% relative abundance in four Palmetto Inland sites, and species of this genus are associated with habitats in the transition zone between freshwater and saltwater, and known to produce detoxification enzymes involved in the degradation of pesticides (Vetriani, Crespo-Medina & Antunes, 2014). It was found in a recent study to be the only genus to increase in relative abundance during a *Wolbachia* infection of *Aedes* mosquitoes (Audsley et al., 2018). Bacterial communities associated with insecticide-resistant mosquitoes differed from those of susceptible mosquitoes, with a greater relative abundance of *Firmicutes* and *Actinobacteria* found in organophosphate-resistant mosquitoes as compared to susceptible mosquitoes (Dada et al., 2018). *Burkholderiales* members are associated with degradation of organophosphate insecticides (fenitrothion) in other insects (Kikuchi et al., 2012), whereas, their role in mosquitoes has yet to be determined. Although we have not determined whether mosquitoes sampled and analyzed from the Palmetto areas were exposed to pesticides in this study, a previous study showed that mosquitoes from west coast Florida including the Palmetto area might have been associated with pesticide resistance (Shin & Smartt, 2016).

The majority of samples from Palmetto sites contained an ASV of *Spiroplasma* (*Mollicutes*), but specifically dominated bacterial communities in two samples (Fig. 3; PI2 and PC4). *Spiroplasma* species have been previously isolated from several mosquitoes, including *Cx. nigripalpus* (Shaikh et al., 1987), and are considered mosquito pathogens (Humphery-Smith, Grulet & Chastel, 1991a; Humphery-Smith et al., 1991b; Chang et al., 2014). Some strains of these bacteria have also been linked to killing males of fruit fly *Drosophila melanogaster* while providing immunity against parasitism by other insects (Xie et al., 2014). It is currently unknown what role it might play in adult *Cx. nigirpalpus* mosquitoes. An ASV classifying to candidatus “*Spironema culicis*”, another spiral-shaped bacterium, also dominated bacterial communities in three samples from Palmetto sites (PI3, PC5, and PC6). This bacterium has been previously found in *Cx. pipiens* (Čechová et al., 2004), but has not been previously reported to be able to dominate the mosquito gut.

The consistent detection of *Thorsellia* spp., although low in abundance, corroborates previous findings of their close association with cross genera mosquito vectors of WNV and SLE viruses (Duguma et al., 2015, 2017b) and malaria parasites (Lindh, Terenius & Faye, 2005; Rani et al., 2009). Although mosquito midgut bacteria usually proliferate post-blood feeding or after sugar meals (Pumpuni et al., 1996), detection of *Thorsellia* spp. in newly emerged mosquitoes, as well as host-seeking adults, suggests a strong association of these bacteria with *Cx. nigripalpus* mosquitoes. These bacteria have been more prevalent in larval mosquitoes (Duguma et al., 2017b) suggesting that they were likely environmentally acquired.

Mosquitoes analyzed in this study were either newly emerged and not blood-fed (Vero Beach mosquitoes) or were seeking hosts at the time they were collected (Palmetto mosquitoes) and were not expected to acquire pathogens from vertebrate blood. However, low proportions of some bacterial ASVs associated with animal and human pathogens or parasites, including *B. cereus*, *Rickettsiella* sp. *Vibrio cholerae*, and *Xanthomonadaceae*, were found in some *Cx. nigripalpus* samples collected from Palmetto sites suggesting that mosquitoes from these sites may have been in contact with vertebrate hosts prior to their collection.

Despite originating from the same mosquito species, life stage, or same geographic region, we observed a remarkably high heterogeneity in the taxonomic profiles of *Cx. nigripalpus* microbiome (Fig. 3). On one hand, there was a tendency of some mosquitoes associating with uneven bacterial species distributions containing one or two ASVs with very high abundance that account for 90% or more of all sequences. The species that showed the tendency to dominate bacterial communities in *Cx. nigripalpus* include known symbionts such as *Asaia* (this study) and *Wolbachia* (Fig. 3, Sample 12; Duguma et al., 2017a). However, this has to be taken with caution and further investigation using *Asaia* and *Wolbachia* gene-specific primers is needed to validate this observation. Other dominant group include *Spironema culicis, Salinisphaera hydrothermalis*, an unclassified *Enterobacteriaceae* ASV, Candidatus Megaira, an uncultured *Burkholderiaceae*, and to a lesser degree, *Pseudomonas* and *Aeromonas* (Fig. 3). The high representation of these taxa in certain mosquito samples but not in others warrant further investigation to understand the underlying biological factors causing this variation.

On the other hand, a relatively high bacterial ASV evenness was observed in many of the samples (Fig. 3). It is evident that many taxa are capable of dominating the mosquito gut environment. The causes or consequences of a high or a low diversity of gut microbial community are not clear. However, in order to harness the gut microbiota for disease vector control or understand their potential impact on pesticide resistance, the heterogeneity in microbial communities in the same species underscores the importance of elucidating the ecological, environmental, genetic, and evolutionary factors that are involved in the assemblage of the mosquito gut community. Our study provides a baseline information to further elucidate the impact of resident microbial taxa in naturally occurring mosquitoes collected from different localities to design symbiotic control such as *Wolbachia*-based vector and disease control approaches.

## Conclusions

Our study indicated microbiota variations among mosquito populations in *Cx. nigripalpus* mosquito disease vector of SLE and WNV. Future research including mosquitoes from broader geographic regions and seasons will assist in better understanding the consequences of microbiota variations in mosquito disease transmission ability and pesticide tolerance (if any) in *Cx. nigripalpus*. Further investigation in examining the factors for the tendency for some taxa to dominate the mosquito gut is required, as they may outcompete taxa such as *Wolbachia* when used as vector-borne disease vector control.

## Additional Information and Declarations

### Data Availability

The following information was supplied regarding data availability: The sequence dataset generated for this study is available at the European Bioinformatics Institute under project accession number PRJEB24029. Data and notebooks are available on GitHub (https://github.com/mwhall/PeerJ_Culex_nigripalpus). Scripts for this publication have been archived in release 1.0 (DOI 10.5281/zenodo.1484552).
